# A Multidisciplinary Intervention in Childhood Obesity Acutely Improves Insulin Resistance and Inflammatory Markers Independent From Body Composition

**DOI:** 10.3389/fped.2020.00052

**Published:** 2020-02-21

**Authors:** Ernst Mayerhofer, Franz Ratzinger, Nina Elvira Kienreich, Annika Stiel, Nadine Witzeneder, Eva Schrefl, Georg Greiner, Christoph Wegscheider, Irene Graf, Klaus Schmetterer, Rodrig Marculescu, Thomas Szekeres, Thomas Perkmann, Martina Fondi, Oswald Wagner, Harald Esterbauer, Matthias Mayerhofer, Stefana Holocher-Ertl, Claudia Wojnarowski, Gregor Hoermann

**Affiliations:** ^1^Department of Laboratory Medicine, Medical University of Vienna, Vienna, Austria; ^2^Department of Neurology and Neurophysiology, Medical Center, University of Freiburg, Freiburg, Germany; ^3^Austrian Social Health Insurance Fund, Vienna, Austria; ^4^FH Wien, University of Applied Sciences, Vienna, Austria; ^5^Division of Hematology and Hemostaseology, Department of Internal Medicine I, Medical University of Vienna, Vienna, Austria; ^6^Ludwig Boltzmann Institute of Osteology, Hanusch Hospital, Vienna, Austria; ^7^Psychology Institute of the University Outpatient Department for Children and Adolescents, Sigmund Freud Private University, Vienna, Austria; ^8^Central Institute of Medical and Chemical Laboratory Diagnostics, University Hospital Innsbruck, Innsbruck, Austria

**Keywords:** childhood obesity, insulin resistance, inflammation, BMI, HOMA-IR, adipokines

## Abstract

Childhood obesity is an increasing health care problem associated with insulin resistance and low-level systemic inflammation, which can ultimately lead to diabetes. Evidence for efficacy of therapeutic intervention programs on the early development of obesity associated sequelae is moderate. This paper investigates the effect of a multidisciplinary short-term intervention program on insulin resistance and metaflammation in childhood obesity. Two hundred and 36 overweight or obese children and adolescents between the ages of 10 and 14 were included in a prospective 5 months intervention study, which included sports, psychotherapy, and nutritional counseling. Primary endpoints were the effects on body mass index standard deviation score (BMI-SDS) and homeostatic model assessment of insulin resistance (HOMA-IR), key secondary endpoints were the levels of C-reactive protein (CRP), leptin, and adiponectin. At baseline, a substantial proportion of participants showed signs of insulin resistance (mean HOMA-IR 5.5 ± 3.4) despite not meeting the diagnostic criteria for diabetes, and low-level inflammation (mean CRP 3.9 mg/l ± 3.8 mg/l). One hundred and 95 participants (83%) completed the program resulting in a significant reduction in BMI-SDS, HOMA-IR, CRP, and leptin and a significant increase in adiponectin (mean change compared to baseline −0.14, −0.85, −1.0 mg/l, −2.8 ng/ml, and 0.5 μg/ml, respectively; *p* < 0.001 each). Effects on BMI-SDS, HOMA-IR, CRP, and adiponectin were largely independent whereas leptin was positively correlated with BMI-SDS and total fat mass before and after intervention (*r* = 0.56 and 0.61, *p* < 0.001 each). Short-term multidisciplinary intervention successfully improved body composition, insulin sensitivity, low-level systemic inflammation, and the adipokine profile in childhood obesity. Our findings highlight the immediate connection between obesity and the pathophysiology of its sequelae, and emphasize the importance of early intervention. Continued lifestyle modification is likely necessary to consolidate and augment the long-term effects.

## Introduction

Recent worldwide trends revealed that previously rising prevalence rates for children with obesity have plateaued in many high-income countries, but accelerated in low- and middle-income countries. In 2016, 124 million children were obese (1). In Austria, prevalence of obesity and overweight was 8 and 18%, respectively, making more than a quarter of the juvenile population overweight or obese (1).

Obesity-associated low-level systemic inflammation (metaflammation) is an important pathophysiological mechanism of atherosclerosis and diabetes and contributes to the early development of insulin resistance (IR) in obesity (2). IR affects up to 25% of non-diabetic children with obesity (3) and amplifies the risk for metabolic syndrome in adulthood (4). A well-established marker for insulin resistance is the homeostasis assessment model for insulin resistance (HOMA-IR) (5), calculated from fasting plasma glucose and insulin, which has high specificity and sensitivity in pubertal adolescents with obesity (6). Children with obesity have approximately doubled HOMA-IR levels compared to normal weight children (7). Slightly elevated levels of C-reactive protein (CRP) represent a surrogate parameter of a systemic low-level inflammation in obesity and have been associated with atherosclerosis, metaflammation, and increasing body fat percentage (8). Adipose tissue is not only excess fat, but serves as metabolically active organ which secretes many different adipokines, in particular leptin and adiponectin (9). Leptin has effects on the central nervous system, muscle, and pancreatic islets, in healthy individuals leading to reduced food intake and increased energy expenditure (9). In obesity, leptin is increased, suggesting leptin resistance (10). Adiponectin acts on the liver and skeletal muscle, reducing glucose production and increasing insulin sensitivity and energy expenditure (9). In children with obesity, adiponectin is reduced in comparison with healthy weight children (11).

Treatment of childhood obesity is essential to prevent sequelae in adulthood. Multidisciplinary therapy programs including the family and targeting behavior change are state-of-the-art for conservative treatment; however, evidence quality for BMI reduction in treatment programs is at most moderate (12, 13). We investigated the short-term effect of a multidisciplinary intervention program on BMI-SDS, IR, and metaflammation and their association in children and adolescents affected by overweight and obesity.

## Participants, Materials and Methods

### Study Design

“Enorm in Form” was a public intervention program conducted by a public Austrian health insurance and the acquired data was scientifically evaluated by the Medical University of Vienna and the Sigmund Freud Private University in Vienna. Written parental informed consent and written assent were obtained for all children. The program conducted was easily accessible, free for the participants, and targeted toward families from low socioeconomic conditions, particularly children from families with migratory backgrounds and/or with low-income. The program was advertised in newspapers, on the internet, and at statutory health insurance physicians' offices. A hotline for counseling and registration was established for 6 h per week. Two hundred and 58 children were screened and 236 were enrolled. Inclusion criteria were age between 10 and 14 years, overweight or obesity (BMI percentile ≥ 90), psychological capability to participate in a group program, willingness, and possibility of child and caregiver to attend the planned activities (see below). Exclusion criteria were secondary obesity of any cause and acute musculoskeletal injuries. Participants with acute or chronic infectious diseases, antibiotic treatment, L-thyroxine or glucocorticoid intake, or other potential interfering medication were excluded from data analysis.

**Figure 1:**
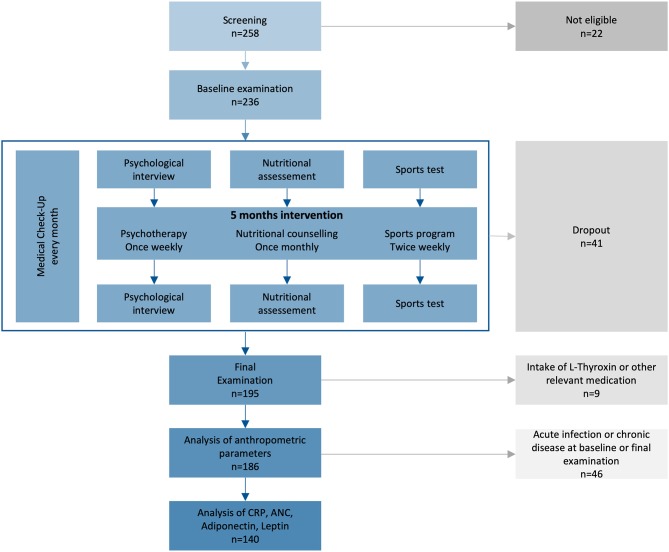
Study plan of the prospective intervention study. From 2014–2017, 236 children from 10–14 years with primary overweight or obesity were included, if they were willing and psychologically and physically capable for the intervention. ANC, absolute neutrophil count; CRP, C-reactive protein.

Intervention groups of 10–12 children were distributed to four ambulatory health care centers where a 5 months intervention was performed. Five cohorts were completed, leading to a total duration of the program of 2.5 years. Each cohort underwent a multidisciplinary program consisting of sports, nutritional counseling, psychotherapy, and medical checkups (Figure 1). The basic exercise program consisted of group gym exercise on two afternoons per week for 90 min each, split up quarterly into warm-up, strength, endurance, and coordination phases aimed to improve general fitness. In addition, we offered various voluntary sports classes 1–2 times a week. The goal of the nutritional counseling was long-term behavioral change of food intake. Single nutritional supervision sessions with the participant and caregiver were performed every 4 weeks for 1 h, starting with measurement of weight, height, and body composition. Nutritionists reviewed participants' food diary and evaluated the food consumption. Consumed food types were marked red, yellow, or green depending on the macronutrient balance and recommended intake frequency. Together with the participants, they worked out individual objectives for the next month and recommended combination of food types, portion sizes, fruits and vegetables, types of drinks, and amounts of macronutrients. To ensure standardization, nutritional counseling was carried out by the same two nutritionists specialized in pediatric counseling over the whole program. Every participant attended weekly 1 h psychotherapy sessions in groups of 6–12 children and an additional monthly 90 min group session with their caregivers. Therapists focused on the effects of relationships on the subjective body experience. Negative social experiences (especially bullying), lack of self-esteem and self-acceptance, the perception of one's own body, the body in relation to the environment, body image, and aspects of sexual identity during adolescence were of particular importance. Together with the therapists, participants made themselves aware of their regulation of food intake. Their goals were the downregulation of the desire for excess food intake, the enhancement of self-esteem, self-acceptance, and life satisfaction.

### Data Acquisition

On baseline and final examinations, we obtained a comprehensive medical history, a physical examination, a test of lung function, an electrocardiogram (ECG), routine laboratory tests with liver and thyroid function parameters, a nutritional knowledge quiz, a fitness test, and a psychological assessment. The fitness test started with a warm-up phase: patients were asked to run 5 min on a treadmill H/P/COSMOS Mercury® with a speed of 5 km/h and no elevation (0°) before measurement of heart rate with a Polar® chest strap. Afterwards they were asked to do as many sit-ups as possible with a 2 kg medicine ball. The psychological assessment included an anamnestic parents-questionnaire (14), an intelligence screening (15), and two self-report forms to assess eating and motion behavior (16) as well as self-esteem of children and adolescents (17). Moreover, parents and children filled in a self-constructed questionnaire to assess behavioral, emotional, und motivational changes. Socioeconomic factors such as type of school attended, occupation of the parents, family income, and mother tongue were collected upon inclusion. Detailed dietary intake was recorded using the validated food frequency questionnaire “What do you eat?” from the German National Health Interview and Examination Survey for Children and Adolescents (18) before and after intervention. Intake of calories was calculated by multiplying estimated amount of foods from the questionnaire with average amounts of macronutrients and calories for each food type. All participants received an ultrasound examination of the thyroid gland, liver, and pancreas, and an ultrasound measurement of subcutaneous and visceral fat thickness before and after the intervention, which was carried out on a Toshiba System SSA66A. Thickness of intraabdominal fat was measured between the back side of the abdominal aorta and the abdominal wall at umbilical height. Subcutaneous fat was measured at three sites: umbilical, subxiphoidal and in the left lower abdomen; the highest value was used. Total body fat was measured by bioelectrical impedance analysis on a Biacorpus RX4000, and data analysis was done with BodyComposition V9.0 software. Monthly medical check-ups included a physical examination and measurement of weight, height, blood pressure, and temperature. Height and weight were obtained by the pediatric nurse and participants were dressed only with pants/skirt and shirt with empty pockets in the pediatric examination room. BMI was calculated by the formula weight/height^2^. We defined overweight as ≥90th, obesity as ≥97th, and extreme obesity as ≥99.5th sex- and age-specific BMI percentile using the recommended reference data from Germany (19). We calculated BMI-SDS from the BMI percentile by using z-score transformation. Laboratory analyses were carried out by Roche (Basel, Switzerland) Cobas 8000 analysis system with C702 analyzer or E602 analyzer according to ISO 15189 accreditation standards as specified in the Supplements. Adiponectin and leptin levels were measured by ELISA according to the recommendations of the manufacturers (BioVendor, Brno, Czech Republic for adiponectin and DRG Diagnostics, Mannheim, Germany for leptin). Elevated fasting plasma glucose was defined as exceeding 5.55 mmol/l (100 mg/dl). OGTT was performed in children with risk factors for impaired glucose tolerance (at least two of the following: type 2 diabetes in first- or second-degree relatives, extreme obesity, signs of insulin resistance or associated alterations (e.g., hypertension, dyslipidemia, elevated liver transaminases, polycystic ovary, or acanthosis nigricans), individuals of Hispanic, Asian, or African origin). We administered 1.75 g glucose per kg body weight (maximum dose 75 g), and measured plasma glucose after 2 h, according to the German guideline for diagnostics and therapy of juvenile overweight and obesity (20). Cut-off levels were as follows: normal glucose tolerance (<7.8 mmol/l; <140 mg/dl), impaired glucose tolerance (<11.1 mmol/l; <200 mg/dl), or diabetes (≥11.1 mmol/l; ≥200 mg/dl). HOMA-IR was calculated by the formula: (fasting insulin [μU/ml] × fasting glucose [mmol/l])/22.5 (5).

### Statistical Analysis

In this interventional study, it was difficult to accurately calculate the sample size a priori because the estimated effects depend on controllable and uncontrollable factors. With BMI-SDS as our main outcome criterion, we expected a similar change as described by Vos et al. (21) (BMI-SDS before intervention: 4.2 ± 0.7, BMI-SDS after intervention: 4.0 ± 0.9). Assuming α = 0.05 and power = 0.9 (two-tailed), a sample size of 178 patients is required (effect size d = 0.244). In addition, a 10% size was added to address any increased variance in the data, resulting in a final sample size of 195 patients fulfilling our intervention program. Due to the pilot character of this study, we did not apply controlling procedures related to the multiple testing problem. Sample size estimation was performed with G^*^Power Version 3.1 (22).

We evaluated the effect of the program on BMI-SDS and used HOMA-IR as the primary endpoint reflecting IR and glucose metabolism. Secondary endpoints were changes of CRP, absolute neutrophile count (ANC), total body fat, intraabdominal fat thickness, leptin, adiponectin, changes in macronutrient and total calorie intake, heart rate after exercise, and maximum number of sit-ups. We calculated mean and standard deviation (SD) for all parameters as descriptive measures. Differences before and after intervention are given as differences of the mean. We used the Spearman product moment correlation for correlation tests, the Wilcoxon signed rank test for comparison of parameters before and after the intervention, and the Chi-square test for association of categorical variables. A mixed-effect repeated measures ANOVA (before vs. after intervention) was fitted (using the lme4 and lmerTest package) modeling the effect of BMI-SDS, CRP, leptin, adiponectin, as well as patient's age, sex, first language (German vs. none-German native language) on HOMA-IR levels. Prior to analysis, numeric parameters, not following a Gaussian distribution (CRP, leptin, adiponectin) were log-transformed. The Bonferroni-Holm correction method for multiple testing was applied with a corrected significance level of 0.05 to define statistical significance. All statistical analyses were carried out in R version 3.6.2 with packages ggplot2, dplyr, childsds (R Project for Statistical Computing, RRID:SCR_001905) on Mac OS X.

## Results

### Baseline Characteristics of the Study Population

Between August 2014 and February 2017, 236 children were enrolled and 195 completed the program (Figure 1, Table S1). Participants were between 10 and 14 years old with balanced age and gender ratios. All participants suffered from overweight, 89% were affected by obesity, and 40% by extreme obesity (Table 1). Sixty-five percent of participants reported one or more failed attempts at weight loss, and 84% had a family history of overweight (Table S3). No children had to be excluded from the sports program due to pathologic lung function test or ECG result. We excluded nine participants (5%) from data analyses because of treatment with L-thyroxine (seven participants) or other (two participants; glucocorticoids, isotretinoin). No participant was using oral contraceptives. Eighty-seven percent of participants reported more than 1 h of exercise per week with a total mean of 3.7 h exercise per week in the nutritional questionnaire. Seventy-one percent of the children spent 2–6 h, 14% more than 6 h and 15% <2 h per day on screen time. The children had 21 different native languages, the most common were German (*n* = 85, 44%), Serbian (*n* = 24, 12%), Turkish (*n* = 22, 11%), Arabian (*n* = 14, 7%), and Other (*n* = 50, 26%).

**Table 1:** Baseline characteristics and change of anthropometric and metabolic parameters during the intervention.

**Participant characteristics**				
All participants^§^	195 (100%)			
Male^§^	93 (48%)			
Female^§^	102 (52%)			
Age [years]^†^	12 ± 1.4			
**Anthropometric parameters**	**Baseline (mean** **±** **SD)**	**After intervention (mean** **±** **SD)**	**Difference**	***p*****-value**
Height [cm]^†^	156.9 ± 10.4	160.3 ±10.2	3.4 (2.2%) ± 1.1	<0.001
Weight [kg]^†^	75.1 ± 18.7	76.7 ± 18.9	1.6 (2.1%) ± 1.9	<0.001
BMI [percentile]^†^	98.7 ± 1.5	98.0 ± 2.1	−0.6 ± 0.2	<0.001
<90 (normal weight)^§^	0 (0%)	1 (1%)	-	-
≥ 90 and <97 (overweight)^§^	22 (11%)	41 (21%)	-	-
≥ 97 and <99.5 (obesity)^§^	96 (49%)	95 (49%)	-	-
≥ 99.5 (extreme obesity)^§^	77 (40%)	58 (30%)	-	-
BMI-SDS^†^	2.44 ± 0.5	2.30 ± 0.5	−0.14 ± 0.1	<0.001
Total fat mass [%]^†^	37.8 ± 6.3	36.0 ± 6.4	−1.8 ± 0.7	<0.001
Intraabdominal fat [mm]^†^	44.0 ±11.7	38.7 ± 11.9	−5.3 (−12%) ± 1.2	<0.001
**Glucose metabolism**				
HOMA-IR^†^	5.5 ± 3.4	4.7 ± 3.4	−0.85 ± 0.4	<0.001
Insulin [μU/ml]^†^	26.1 ± 14.9	21.9 ± 14.2	−4.20 ± 1.5	<0.001
C-peptide [ng/ml]^†^	3.4 ± 1.1	3.1 ± 1.0	−0.33 ± 0.1	<0.001
Glucose [mmol/l]^†^	4.7 ± 0.4	4.7 ±0.4	−0.02 ± 0.0	NS
HbA1c [%]^†^	5.3 ± 0.3	5.3 ± 0.3	0.0 ± 0.0	NS
HbA1c [mmol/mol]^†^	34.1 ± 3.5	34.1 ± 3.6	−0.01 ± 0.4	NS
**Metaflammation**				
CRP [mg/l]^†^	3.93 ± 3.8	2.9 ± 13.1	−1.02 ± 0.42	<0.001
Absolute neutrophil count^†^	3.87 ± 1.6	3.75 ± 1.3	−0.12 ± 0.13	NS
Leptin [ng/ml]^†^	15.0 ± 8.0	12.2 ± 7.9	−2.8 ± 0.9	<0.001
Adiponectin [μg/ml]^†^	7.7 ± 2.3	8.2 ± 2.3	0.5 ± 0.3	<0.001

*Sex and age were equally distributed between males and females from 10 to 14 years. All participants were affected by overweight or obesity. Total fat mass was obtained via bioimpedance measurement, intraabdominal fat measured by ultrasound examination*.

*NS, not significant*.

§ *Number of participants and ratio (%)*.

† *mean and standard deviation values*.

### Attendance to the Multidisciplinary Intervention

The dropout rate was 17% (41/236; Table S2 shows dropout reasons). Data of dropped-out subjects were excluded from analysis. Differences in the attendance rate were observed for children that completed the program. Eighty-eight percent of all nutritional counseling units were attended, giving it the highest attendance rate, and 75% of participants participated in all offered units. Psychotherapy was attended at a mean rate of 76%. Sports program had a mean attendance rate of 72%, and 60% of all participants attended more than 75% of all offered sports units. Seventeen participants (9%) participated in additional sport units.

### High Rate of Insulin Resistance and Subclinical Inflammation in Non-diabetic Obese Children

First, we analyzed glucose metabolism and IR of the cohort at baseline. None of the children showed elevated fasting glucose. OGTT was performed in 115 children, and impaired glucose tolerance was only found in 4 participants (3.5%). In contrast, insulin and C-peptide levels were elevated in 45% and 14% of participants (Table 1). This corresponds to a mean HOMA-IR of 5.51 (SD 3.4), indicating a considerable proportion of IR in non-diabetic children with overweight or obesity (Table 1). A poor correlation between baseline BMI-SDS and HOMA-IR was observed (*r* = 0.38; *p* < 0.001). While the mean level of the inflammation marker CRP was within the reference range, 28% of the children had CRP levels >0.5 mg/l without clinical signs of infection. Interestingly, correlation between baseline CRP and BMI-SDS and baseline CRP and HOMA-IR was rather low (*r* = 0.30, *p* < 0.001 and *r* = 0.04, *p* = 0.64, respectively). In the subgroup of children with migratory background, baseline BMI-SDS and HOMA-IR showed a not-significant trend to be higher than in the subgroup of children with German as native language (BMI-SDS: 2.53 ± 0.47 vs. 2.32 ± 0.47, *p* > 0.05; HOMA-IR: 5.7 ± 3.2 vs. 5.2 ± 3.6, *p* > 0.05).

### Effects of the Short-Term Multidisciplinary Intervention Program on Body Composition, Physical Fitness, Nutrient Intake

**Figure 2:**
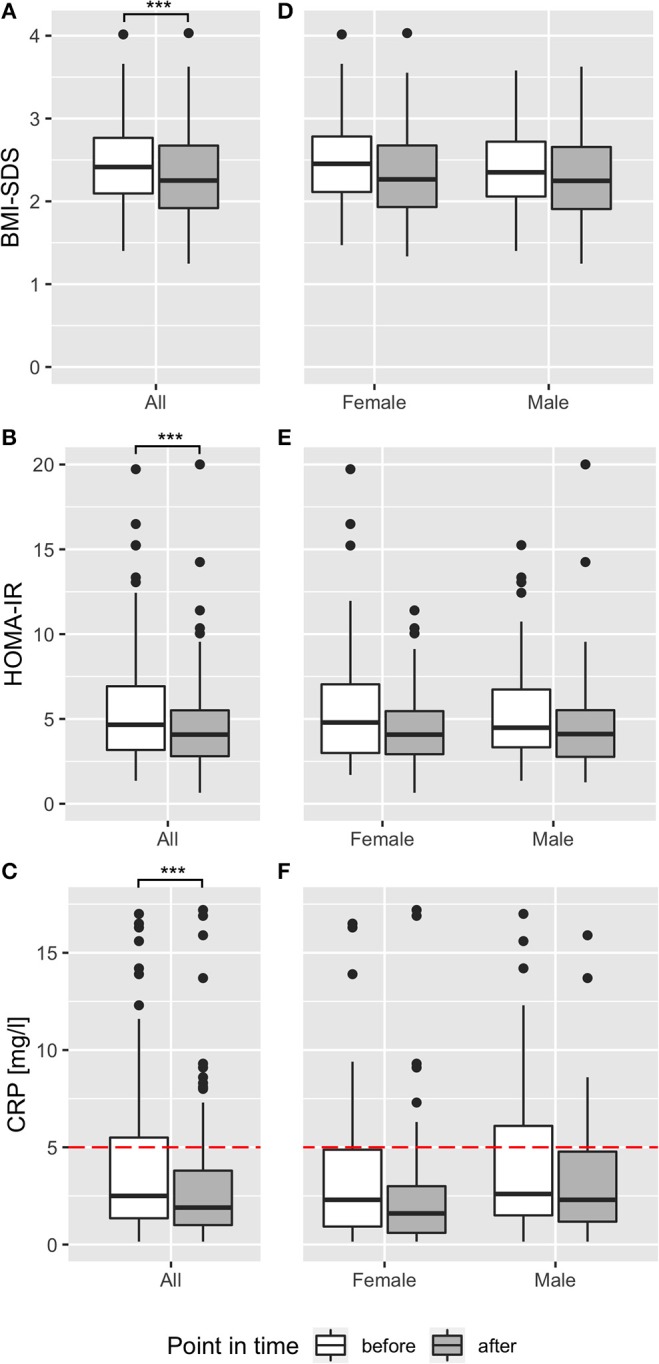
Effect of multidisciplinary intervention on body composition, insulin resistance, and inflammation. Effect of intervention on BMI-SDS **(A,D)**, HOMA-IR **(B,E)**, and CRP **(C,F)** before (white) and after (dark gray) multidisciplinary treatment are shown as boxplots for the whole participants' cohort **(A–C)** and stratified for sex **(D–F)**. Red dotted lines show reference ranges, where applicable. ***p < 0.001.

BMI-SDS was used to judge efficacy of the program. Mean BMI-SDS was significantly reduced by 0.14 from 2.44 before to 2.30 after intervention (*p* < 0.001, Figure 2A, Table 1). Seventy-nine percent of participants showed a reduction of BMI-SDS (by a mean of 0.21), whereas BMI-SDS stayed the same or increased in 21% of participants. Sex-specific analysis showed the same effects of the intervention on BMI-SDS in boys and girls (Figure 2D). Thirty-four percent of participants decreased BMI-SDS by 0.2 or more. BMI-SDS was significantly reduced both in children with migratory background and in children with German as native language (BMI-SDS change −0.12 vs. −0.15, *p* < 0.001 for both), and the reduction was existent in all baseline BMI-SDS quartiles (Table S7). In summary, the multidisciplinary intervention significantly reduced BMI-SDS irrespective of gender, migratory background or initial BMI-SDS. Equally in boys and girls, mean total body fat percentage was reduced by 1.8% from 37.8% before to 36% after intervention (*p* < 0.001), and intraabdominal fat thickness was lowered by 5.3 mm (13%) from 44.0 mm before to 38.7 mm after intervention (*p* < 0.001; Figure S1, Table 1). Results of the fitness test showed a significant improvement in cardiovascular and physical fitness of the children. The mean heart rate after 2 min running decreased by 4.5% from 144.3 ± 22.6 bpm before intervention to 137.8 ± 20.8 bpm after intervention (*p* < 0.001). The mean number of maximum sit-ups possible per patient increased by 30% from 27.3 ± 15.0 to 33.5 ± 18.9 (*p* < 0.001). These effects were found in both genders, but more pronounced in boys than in girls (heartrate reduction 6.6 vs. 2.4% and increase in maximum sit-ups 37 vs. 13%, respectively). The self-reported average daily intake of macronutrients fat, protein and carbohydrates was significantly decreased by 34, 20, and 28%, respectively (*p* < 0.001 for all), while fiber intake was unchanged (*p* = 0.35). Calorie intake was significantly reduced by 29% (*p* < 0.001). Estimated daily calorie intake was decreased by 630 kcal from 2,160 kcal before intervention to 1,530 kcal after intervention (Table S8). These effects were existent in both genders, but more pronounced in girls than in boys (37 vs. 22% calorie reduction, respectively).

### Short-Term Multidisciplinary Intervention Reduced Insulin Resistance and Metaflammation

HOMA-IR was used as the primary endpoint to analyze effects of the multidisciplinary intervention on glucose metabolism. HOMA-IR was significantly decreased by 0.86 from 5.5 before to 4.7 after intervention (*p* < 0.001; Figure 2B, Table 1). Sixty-eight percent of all participants decreased HOMA-IR levels during intervention, reducing HOMA-IR levels in this group by 2.03. Compared with recently published reference ranges for children with obesity (7), the ratio of participants over the 90th percentile of HOMA-IR decreased from 53 to 36% within the intervention. Regarding sex-specific differences, HOMA-IR was reduced in boys and girls, but the reduction was more pronounced in girls (reduction by 1.12 from 5.6 ± 3.31 to 4.48 ± 2.25; *p* < 0.02) than in boys (reduction by 0.58 from 5.41 ± 3.45 to 4.83 ± 4.37; *p* = 0.003), pointing to better therapy response of girls in terms of glucose metabolism (Figure 2E). Similarly, the percentage of participants over the 90th percentile of HOMA-IR decreased within the intervention from 51 to 31% in girls and from 57 to 42% in boys. Mean HOMA-IR levels, grouped by age, showed a reduction in every age with different baseline levels, peaking at age 12 (Figure S2). Decrease in HOMA-IR was equally observed both in children with migratory background and in children with German as native language (−0.87 vs. −0.83, *p* < 0.001 for both). To rule out a potential confounding effect of puberty, effects of the intervention program on HOMA-IR were assessed in patients who were after onset of puberty before and after intervention. Mean HOMA-IR levels before/after intervention in the subgroup of girls who had menarche already before intervention (*n* = 44) and in the subgroup of boys with SHBG values <50 nmol/l before intervention (*n* = 69), indicating Tanner stage II (23), were compared and were significantly different (difference in HOMA-IR was −0.72 for girls, −0.57 for boys; *p* < 0.05 for both; Figure S4). Furthermore, correlations of HOMA-IR difference with sex hormone differences before and after intervention yielded no correlation for LH, FSH, DHEAS, and testosterone (*r* = −0.02, *r* = 0.11, *r* = 0.09, and *r* = 0.04; *p* > 0.05 for all; Figure S5), pointing to reduction in insulin resistance independent from pubertal state. In line with the HOMA-IR reduction, serum insulin levels and C-peptide levels were reduced after intervention (Table 1; Figure S1). All participants had normal glucose levels before, and one participant had slightly (5.77 mmol/l) elevated glucose after the intervention. HbA1c levels were normal for all participants except one (6.1%, 43 mmol/mol) after intervention. In summary, a significant reduction of IR in all sex and age groups with a pronounced effect in girls was observed for non-diabetic children with overweight and obesity after multidisciplinary intervention. To rule out masked impaired glucose tolerance or diabetes in participants without OGTT, we analyzed the effect of treatment in the subgroup of participants who underwent OGTT. Comparison of baseline measurements showed no significant difference (*p* > 0.05) except for BMI-SDS (since this was a screening criterion for performing OGTT) and leptin (which was positively correlated with BMI-SDS). Furthermore, treatment effects were equal, indicating no difference between participants with OGTT and the whole sample (Table S4).

We excluded 34 (17%) participants suffering from acute infections and 12 patients suffering from chronic diseases (bronchial asthma *n* = 5, recurring bronchitis *n* = 4, recurring otitis *n* = 1, psoriasis *n* = 1, recurring urinary tract infections *n* = 1) from analysis of CRP and ANC. Of the remaining children, 29% had CRP levels > 5 mg/l before the intervention, and 18% at final examination. Two-thirds of participants analyzed and included in analysis both before and after intervention decreased CRP levels within the program. In total, CRP levels were effectively reduced by 1.02 mg/l from 3.93 before to 2.90 after intervention (*p* < 0.001; Table 1, Figure 2C). Sex-specific analysis revealed lower levels in girls than in boys, after intervention (2.54 ± 3.16 mg/l vs. 3.33 ± 3.05 mg/l, respectively) and at baseline (3.74 ± 3.96 mg/l vs. 4.14 ± 3.67 mg/l, respectively; Figure 2F). There was a not-significant trend in ANC to a decrease by 0.12 G/l from 3.87 before to 3.75 after intervention in all children (Table 1), gender-specific analysis revealed this trend was only existent in girls (reduction by 0.20 G/l in girls vs. no reduction in boys; *p* > 0.05 for both; Tables S5, S6). When we studied adipokines as additional markers of metaflammation, leptin was significantly reduced by 2.8 ng/ml after intervention (12.2 ± 7.9 ng/ml) compared to baseline (15.0 ± 8.0 ng/ml; Table 1; Figure 4B). This reduction was found both in boys and girls, however girls had 2–3 ng/ml higher leptin levels before and after intervention than boys (Tables S5, S6). Adiponectin was significantly increased by 0.5 μg/ml after intervention (8.2 ± 2.3 μg/ml) compared to baseline (7.7 ± 2.3 μg/ml; Table 1; Figure 4C). This increase was also existent in both genders, but girls had 0.5 μg/ml lower adiponectin levels than boys before and after intervention (Tables S5, S6).

### Reduction of Insulin Resistance and Metaflammation Is Largely Independent From Body Composition

**Figure 3:**
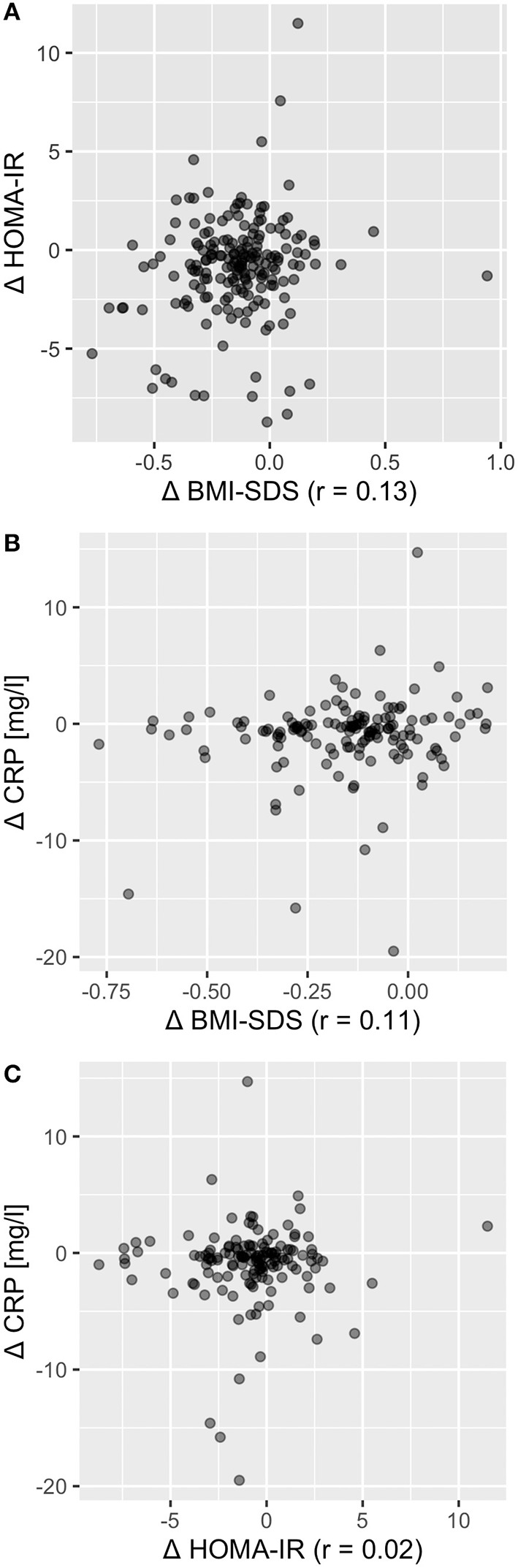
Change in BMI-SDS, HOMA-IR, and CRP were largely independent from each other. Correlation coefficient for Δ BMI-SDS with Δ HOMA-IR was *r* = 0.13 **(A)**, for Δ BMI-SDS with Δ CRP *r* = 0.11 **(B)**, and for Δ HOMA-IR with Δ CRP *r* = 0.02 **(C)**, indicating independency of change in BMI, insulin resistance, and metaflammation.

**Figure 4:**
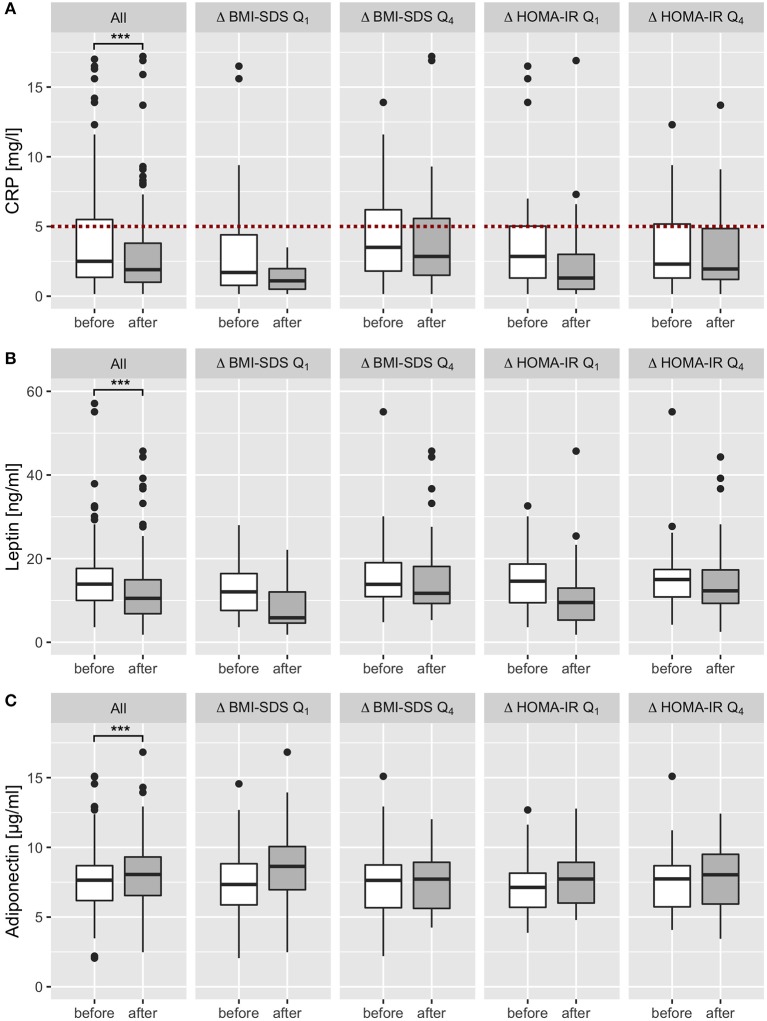
Effect of multidisciplinary intervention on biomarkers of metaflammation. After exclusion of all patients with acute infectious and chronic diseases, CRP, leptin and adiponectin levels were analyzed before (white) and after intervention (dark gray) and stratified by the effect on BMI SDS and HOMA IR (subgroup analysis of the first and last quartiles of Δ BMI SDS and Δ HOMA IR). CRP levels were widely within the reference range (<5 mg/l; red dotted line) and showed a significant decrease after intervention in all patients **(A)**. Leptin levels decreased in all patients significantly; subset analysis showed a marked decrease in first quartiles (Q1) of BMI-SDS and HOMA-IR changes, and only a slight decrease in patients who showed no therapy success or aggravation (Q4) **(B)**. Adiponectin levels increased in all patients significantly, subset analysis showed a marked increase in the first quartile (Q1) of BMI-SDS variation, a slight increase in the first quartile of HOMA-IR variation, and no change in the last quartile of BMI-SDS and HOMA-IR change **(C)**. ***p < 0.001.

BMI-SDS and HOMA-IR were poorly associated at baseline (*r* = 0.38; *p* < 0.001) and after intervention (*r* = 0.45; *p* < 0.001). To evaluate interdependency of body composition and IR, we calculated correlation of BMI-SDS and HOMA-IR variation, which showed no relation (*r* = 0.13; *p* = 0.09; Figure 3A). Furthermore, correlation of HOMA-IR difference with total body fat and intraabdominal fat difference was also poor (*r* = 0.17 and *r* = 0.12; *p* > 0.05 for both). Despite a positive effect on the overall cohort, not all participants responded with BMI-SDS and/or HOMA-IR reduction. Therefore, we divided participants into subsets by BMI-SDS and HOMA-IR quartiles and used them for further analyses. The first and last quartile (Q_1_ and Q_4_) subsets represent the fraction of participants who showed the best therapy success (Q_1_) and worst success/aggravation (Q_4_) in regard to BMI-SDS and HOMA-IR. By calculating the change of the opposing parameter in Q_1_ and Q_4_ of both parameters, we found that BMI-SDS and HOMA-IR were widely independent (Figure S3), which is consistent with the correlation finding. Also, there was no correlation of BMI-SDS and HOMA-IR change with CRP change (*r* = 0.11 and *r* = 0.02; Figures 3B,C), although CRP decrease in BMI-SDS Q_1_ was greater compared to that in Q_4_ (−1.9 mg/l vs. −0.7 mg/l; Figure 4A). There was no correlation of ANC with BMI-SDS or HOMA-IR at baseline, after intervention or for the change of the parameters (*r* < 0.3 and *p* > 0.05 for all correlations). Leptin levels showed a fair positive correlation with BMI-SDS before and after intervention (*r* = 0.56 and 0.64, respectively; *p* < 0.001 for both), and leptin change had a poor correlation with BMI-SDS change (*r* = 0.27; *p* < 0.05). Leptin levels correlated better with fat mass (*r* = 0.61 before, *r* = 0.71 after intervention, *p* < 0.001 for both), and correlation for change of leptin with change of fat mass was rather poor (*r* = 0.30, *p* < 0.001). Further correlation tests yielded poor correlation for change of leptin with change of HOMA-IR, leptin levels before/after intervention with HOMA-IR before/after intervention (*r* = 0.21, *p* < 0.05; *r* = 0.4, *p* < 0.001; *r* = 0.32, *p* < 0.001). Change of adiponectin showed a poor inverse correlation with change of BMI-SDS (*r* = −0.36, *p* < 0.001) but not with change of HOMA-IR (*r* = −0.1, *p* = 0.25).

sWe used a linear mixed-effect model evaluating the effect on HOMA-IR to evaluate independency of variables and evaluate confounders. No association was observed for age (*p* = 0.75), gender (*p* = 0.96), native language (*p* = 0.45), BMI-SDS (*p* = 0.07), CRP (*p* = 0.06), and adiponectin (*p* = 0.16), but a significant association was observed for leptin (*p* < 0.001). Finally, we compared baseline characteristics and attendance rates between the BMI-SDS and HOMA-IR quartiles and found no difference for age, gender, native language, and attrition rate in psychotherapy or nutritional counseling between first and last quartile (*p* > 0.05 for all).

## Discussion

The program was successful in the short-term treatment of childhood obesity by improving body composition. The dropout rate of 17.4% was low, compared to a dropout rate of 27–73% in other pediatric weight management programs (24). Within the intervention period of 5 months, 80% of the participants responded to the therapy in terms of BMI-SDS and two third in terms of IR and metaflammation. Therapy responders lowered BMI-SDS by 0.21 and HOMA-IR as marker for IR by 2.03, raising the ratio of participants without IR to two thirds after intervention. This is comparable with other studies, which achieved BMI-SDS decreases between 0.12 and 0.4 (21, 25, 26), and HOMA-IR decreases from 0.1 to 2.4 within an amount of time ranging from 8 weeks to 1 year (27, 28). A recent meta-analysis of interventions shows mean BMI-SDS reductions of 0.02 and 0.13 for interventions less than and longer than 6 months, respectively (12). Thus, the efficacy of the 5 months program was respectable compared to similar studies. Of course, the majority of participants were still obese after completion of the program. Given that additional gain of weight and increase of BMI is the typical natural history of obesity (29), we nevertheless believe that even a modest reduction of BMI-SDS and HOMA-IR is clinical meaningful. BMI in adolescence correlates with BMI in adulthood and is a strong predictor of glucose utilization in early adulthood (30). Childhood obesity also increases the risk of diabetes and abdominal obesity in adulthood independently of each other (31), and insulin resistance amplifies the risk for metabolic syndrome in adulthood (4). Certainly, the changed lifestyle must be continued in order to maintain or enhance the effects long-term. By providing measurable body improvements and communication with other children affected by obesity, the program might have been a motivational factor for continued lifestyle improvement. Skills gained during the intervention (nutritional knowledge, self-esteem, behavioral change of food intake) may help to fight obesity. There is recent evidence that weight-loss after behavioral intervention programs can be maintained long-term, and compared to untreated controls, a 3 months program has sustained impact on biometrics and physical activity after 1 year (12, 32, 33). To promote ongoing profound changes in diet, physical exercise and behavior, we offered easily accessible continuing classes for the children after the program. Despite the overall success, not all children showed improvements of BMI-SDS or HOMA-IR after the intervention program. We, however, did not identify a major predictor for treatment response in our cohort. In particular, no effect of gender, age, or migratory background was observed.

The design of our intervention program showed similarities and differences to other comparable programs. Like other, we considered family integration as crucial, especially in children, who are more dependent on their family than adolescents (34, 35). We therefore integrated the caregivers in every session of nutritional counseling (every 4 weeks) to create a less obesogenic environment at home. Before every session, body fat percentage was measured by bioimpedance analysis, giving the participants objective feedback about the current body composition. According to the nutritionist's subjective experience, this was one of the main motivators for adhering to their recommendations. Unlike other behavioral interventions, our psychotherapy group sessions focused on the subjective body experience, negative social experiences, lack of self-esteem and self-acceptance, which are the consequences of obesity and not the causes. One additional group session together with the caregivers was conducted every month to make them aware of the psychological problems of the children and the importance to treat obesity. In the medical check-ups every 4 weeks, patients and caregivers could catch up about the program and address symptoms or problems regarding the intervention with the physician, who was the main contact person for the program. This might have been an important factor for continuing the program and for our low drop-out rate. Our study focused on children in age of 10–14 years. The age group was chosen due to the lack of other public intervention programs for this age group in Vienna; an additional aspect was that mixed gender sport classes were still feasible in this cohort. Due to the easily accessible character and without any costs for the participant families, more than half of the children had migratory background. We observed a not-significant trend to higher baseline BMI-SDS and HOMA-IR in children with migratory background, but BMI-SDS and HOMA-IR decrease was equally present in both groups, indicating the same treatment response in both groups. In the ANOVA analysis, the native language of the children had no effect on HOMA-IR. Therefore, we think our study protocol is a working intervention in childhood obesity, especially in the changing society with more and more migrants in Europe.

Conservative treatment of obesity has been shown to reduce BMI-SDS, IR, and CRP in other programs (21, 36–39), but the association and development of BMI, IR, inflammation, and adipokines in the treatment of obesity are not fully understood. The rapid change in IR observed during the short-term intervention, even before substantial BMI-SDS change, speaks for the direct association of intervention and therapy effect on metabolism and underlines the importance of treatment. While the majority of participants with extreme obesity (40%) showed signs of IR by means of elevated HOMA-IR levels, levels of fasting glucose, HbA1c, and OGTT were normal before and after intervention. These findings indicate that there is already subclinical IR in non-diabetic children with overweight and obesity. Recent research suggests that HOMA-IR is a robust parameter for detecting impaired insulin sensitivity in individuals at high risk for having IR (40), has high specificity and sensitivity compared with OGTT in pubertal adolescents with obesity (6) and directly correlates to the number of metabolic syndrome components (41). In conclusion, HOMA-IR may be sufficient as an early-stage monitoring parameter in non-diabetic children and adolescents with obesity. Our study showed that IR in non-diabetic children and adolescents is sensitive to short-term lifestyle intervention, shown by the decrease of HOMA-IR in our collective results. IR was shown to be influenced by puberty, and obesity itself is recognized as a modulator of pubertal progress, as it causes earlier onset of puberty in girls, maybe also in boys (42, 43). HOMA-IR levels in children and adolescents physiologically vary with age in all children and are higher in children and adolescents with obesity, peaking between the ages of 13 and 15 before returning to normal levels at the end of puberty (7). Cut-off values for HOMA-IR are therefore much higher in pubertal compared with prepubertal individuals with obesity (44). After our intervention, mean age- and sex-specific HOMA-IR values were lowered and compared with recent reference ranges that take puberty into account, most of the children were under the 90th percentile for children with obesity (7). Therefore, we believe that if physiological age-dependent changes in IR might interfere with our measurements, we underestimated the effect of the treatment on insulin resistance. This idea is also supported by the decrease in HOMA-IR being visible in all ages observed, which is much higher than the physiological age-specific change (7). Sex hormones (FSH, LH, DHEAS, and testosterone) have shown to increase during puberty (45, 46) and are therefore a suitable parameter for observing the progression of pubertal state (47). We found no association between the changes of serum sex hormones with the differences in insulin resistance. These results have strengthened our confidence that the decrease in IR was a consequence of the treatment, and onset or progress of puberty was no major confounder.

The missing correlation between BMI-SDS change and HOMA-IR change is somehow surprising. Since BMI does not reflect a shift from fat toward muscle tissue in body composition, we also correlated HOMA-IR with total body fat and intraabdominal fat, which showed no better association. This suggests that the decrease in IR, caused by exercise/nutritional changes, is independent from body composition in our study and there might be a different underlying mechanism for IR in obesity. These findings correlate well with other data, including an 8 weeks controlled exercise-intervention program for 10–17 years old mostly females that showed association of change in cardiovascular fitness with decrease in IR, but not BMI-SDS (27). In contrast, some studies showed association of BMI-SDS and HOMA-IR differences after longer interventions (48, 49). Furthermore, recent research shows that liver fat, but not total body or visceral fat, is an independent determinant of IR in adolescents (50), pointing to the liver as a pivotal organ in the equation. Another possibility is that the short-term effect of the exercise increases glucose uptake into the muscle, leading to rapid drop of the insulin secretion and lower HOMA-IR values.

While CRP is mostly used as sensitive acute-phase parameter indicating inflammation, baseline serum levels in adults are influenced by age, smoking, lipids, oral contraceptives, and cardiovascular diseases (51, 52). When excluding acute infections, CRP has been shown to be elevated in children with obesity (53), to be associated with hyperlipidemia (54) and to predict IR (55). Moreover, CRP levels predict obesity after 1–6 years of follow-up (53, 56), providing a potential therapy goal in short-term treatment. Almost one-third of the participants in our study had elevated CRP levels, which is in line with other studies that report between 1.5 and 15 mg/l for participants with obesity (39, 57, 58). The observed decrease in overall CRP serum levels after the intervention was statistically significant and especially larger in the quartiles with great changes of BMI-SDS or HOMA-IR, indicating treatment response. This supports the hypothesis that obesity itself contributes to a low-grade systemic inflammation and furthermore is reversible by lifestyle modification (39, 59). There is strong evidence that obesity-triggered metaflammation is a substantial influencing factor, especially for the development of IR (60). Furthermore, the association of obesity and cardiovascular incidents has not only been statistically demonstrated, but has also been reinforced by the fact that certain coagulation factors are increased in obesity (61). Moreover, coagulation and inflammatory systems share a common ancestry and many pathways of interrelations have already been demonstrated (62, 63). Subsequent evaluations in our study population could also reveal possible relations with other metaflammation parameters and the coagulation system. ANC, another unspecific marker of inflammation, has been association with BMI in obese children (64, 65) and might contribute to atherosclerosis in adults (66). We, however, did not observe an effect of the intervention on the ANC. Regarding the adipocytokines leptin and adiponectin, we found leptin reduced after intervention and a fair positive correlation with BMI-SDS and total body fat. This is in accordance with other studies, which found leptin increased in children and adults with obesity and a significant pre/postintervention decrease compared with control groups (67). The physiological higher percentage of adipose tissue in girls might explain the overall higher leptin levels in girls. In the ANOVA analysis, leptin levels had significant influence on HOMA-IR, indicating an important role of leptin in IR. The metabolic change of adipokine expression in fat tissue induced by the intervention might play a significant role in the decline of IR. Obesity is associated with lowered adiponectin, and increasing as well as decreasing levels of adiponectin have been found in pediatric intervention studies (68). Physical exercise alone was found sufficient to reduce leptin and raise adiponectin levels (68). Thus, the observed effects on adipokines in our study are in line with reports from other programs and indicate an effect of the intervention on the adipose tissue and associated metaflammation.

Our work clearly has some limitations. First, the study is missing a control group. Since there has already been evidence of effectiveness of multidisciplinary intervention on obesity, we decided to treat all eligible children rather than taking blood samples from children without intervention. Since untreated obesity and associated IR are very likely to persist during adolescence and into adulthood (21, 69), and because insulin resistance in childhood obesity increases the risk of metabolic syndrome in adulthood (4), it is highly likely that the observed improvements are, in fact, effects of the treatment. A control group with healthy weight kids or a crossover study design would be a possibility for future studies to evaluate the beneficial effect of nutritional counseling, sports and psychotherapy to prevent obesity. In line with this, since the program was advertised publicly and only families actively answering to the advertisements were screened for entering the study there might be a relevant selection bias toward highly motivated participants or families. This bias is particularly relevant as no control group has been enrolled and some of the benefits observed may be due to high motivation of families. In particular, the relatively low dropout rate observed in the program might be linked to this selection bias. Likewise, while the majority of dropouts occurred because of the participants' decision to leave the study, 13 participants (5.5%) were excluded from the program by the principal investigator because of poor adherence. While this was necessary for maintaining the compliance of the rest of the group, it might have biased the results. Furthermore, we did not carry out OGTT in all participants. The applicable guidelines indicate OGTT in the presence of well-defined risk factors. Since OGTT is a strenuous procedure requiring compliance, it was not carried out in all participants. To rule out a potential bias, we confirmed the results from the whole sample in the subgroup of participants with OGTT. Regarding the nutrition objectives, we relied on self-reporting and cannot objectively confirm sustainable change of eating habits. While we used a validated food questionnaire, the impressive reduction of macronutrient and calorie calculation was only a rough estimation of the real intake and might not only show a change in eating habits, but may also be affected by improvements in nutritional knowledge leading to a bias of self-reporting. Finally, the observation period of 5 months shows only short-term effects of the multidisciplinary treatment on body composition and IR. This might be the reason for the missing correlation in BMI-SDS and IR, since BMI-SDS changes were not as pronounced as improvements in IR. Long-term effects of the intervention and sustainability of the improvements cannot be interfered from our data. Clearly, additional studies with increased duration of follow-up are needed to evaluate long-term efficacy of the program.

In summary, the multidisciplinary short-term intervention program “Enorm in Form” significantly improved body composition, insulin resistance and metaflammation in obese children and adolescents of which a large proportion had migratory background. The observed effect size is comparable to other intervention programs and the dropout rate was low. Our findings highlight the immediate connection between obesity and the pathophysiology of its sequelae, and emphasize the importance of early intervention. However, continued lifestyle modification is likely necessary to consolidate and augment the effects of the program in long-term.

## Data Availability Statement

The datasets generated for this study are available on request to the corresponding author.

## Ethics Statement

The studies involving human participants were reviewed and approved by Medical University of Vienna and the Sigmund Freud Private University in Vienna. Written informed consent to participate in this study was provided by the participants' legal guardian/next of kin.

## Author Contributions

CWo, MF, HE, MM, and GH planned the study. CWo recruited patients and supervised the study. IG, NK, AS, CWe, and EM collected the clinical data. SH-E performed psychological tests. ES, NW, GG, KS, RM, TS, TP, OW, and MM processed samples, performed laboratory analysis or provided test results. EM, IG, and GH performed literature search. EM and FR analyzed the data and generated the figures. EM and GH wrote the manuscript. All authors revised and approved the manuscript.

### Conflict of Interest

The authors declare that the research was conducted in the absence of any commercial or financial relationships that could be construed as a potential conflict of interest.
