# Supplementary material for: A Multidisciplinary Intervention in Childhood Obesity Acutely Improves Insulin Resistance and Inflammatory Markers Independent From Body Composition
Source: Front Pediatr. 2020 Feb 21;8:52. doi: 10.3389/fped.2020.00052 (PMC7047334; doi:10.3389/fped.2020.00052)
Supplement: Supplementary file 1 [file Data_Sheet_1.docx]

Supplementary Material

**Supplementary Methods**

Laboratory analyses were carried out as following: : CRP by CRPL2 latex test kit (lower limit of detection [LLOD]: 1.0 mg/l, intra-assay coefficient of variation [CV]: ≤ 2%), insulin by Elecsys Insulin test kit (LLOD: 0.2 μU/ml, intra-assay CV: < 2%), C-peptide by C peptide ECLIA test (LLOD: 0.010 ng/ml, intra-assay CV: < 5%), glucose by the Gluco quant Glucose HK test (second generation, LLOD: 0.555 mmol/l, 10.0 mg/dl, intra-assay CV: < 2%), HbA1c% (LLOD: 3.5%, intra-assay CV: 1.15%) by the HPLC (Variant II Turbo, Bio-Rad, California), sex hormones by Elecsys test kit (for FSH and LH: LLOD: 0.1 mIU/ml, intra-assay CV: < 5%; for testosterone: LLOD: 0.025 ng/ml, intra-assay CV 2.5-18.1%; for Dehydroepiandrosterone [DHEAS]: LLOD: 0.003 μmol/L, intra-assay CV < 3%).

**Supplementary Tables**

| Reason for exclusion | Number of patients |
| --- | --- |
| Time constraints with school afternoon classes | 11 |
| Caregivers could not participate because of various reasons | 6 |
| Severe psychiatric comorbidities | 5 |

Table S1. Reasons for exclusion of 22 patients of the program.

| Dropout reason | Number of patients |
| --- | --- |
| Discontinued without giving reasons | 14 |
| Poor adherence | 13 |
| Psychological problems | 4 |
| School problems | 3 |
| Not enough time | 3 |
| Moved away | 2 |
| Personal problems within the group | 1 |
| Injury | 1 |

Table S2. Reasons for drop-out of 41 patients from the program.

| Characteristic | Patients | Ratio |
| --- | --- | --- |
| All | 195 | 100% |
| Gender | | |
| Male | 93 | 47.7% |
| Female | 102 | 52.3% |
| Age (years) | | |
| 10 | 53 | 27.2% |
| 11 | 47 | 24.1% |
| 12 | 43 | 22.1% |
| 13 | 34 | 17.4% |
| 14 | 18 | 9.2% |
| BMI Percentile |  |  |
| >= 90 and < 97 (overweight) | 22 | 11.3% |
| >= 97 and < 99.5 (obesity) | 96 | 49.2% |
| >= 99.5 (extreme obesity) | 77 | 39.5% |
| Overweight of family members (one of: mother, father, grandparents, siblings) | 164 | 84.1% |
| Mother | 95 | 48.7% |
| Father | 71 | 36.4% |
| Both parents | 38 | 19.5% |
| Siblings | 43 | 22.1% |
| Grandparents | 91 | 46.7% |
| Mother, father, siblings and grandparents | 4 | 2.1% |
| Pregnancy diabetes of the mother | 27 | 13.8% |
| Patient has always been heavier than other children | 43 | 22.6% |
| Significant weight gain at: age 7 ± 2.6 years (5 ± 2.4 years ago) | 147 | 77.4% |
| Weight loss attempts before | 126 | 64.9% |

Table S3. Sex, age and BMI percentile distribution of patients upon inclusion in the study. Age and sex were evenly distributed (m/f between 10-14 years), while almost 90% of patients suffered from obesity and almost 40% of them suffered from extreme obesity.

| **Patient characteristics** | | | | | | |
| --- | --- | --- | --- | --- | --- | --- |
| All patients ^§^ | 115 (100%) | | | | |  |
| Male ^§^ | 50 (43%) | | | | |  |
| Female ^§^ | 65 (57%) | | | | |  |
| Age [years] ^†^ | 12 (2.32) | | | | |  |
| **Anthropometric parameters** | **Baseline  (mean ±SD)** | **After intervention (mean±SD)** | **Difference (mean±SD)** | **p value** | |  |
| Height [cm] ^†^ | 157.5 ± 10.9 | 160.9 ± 10.4 | 3.5 ± 1.5 | < .001 | |  |
| Weight [kg] ^†^ | 78.6 ± 21.0 | 80.3 ±21.0 | 1.6 ± 2.9 | < .001 | |  |
| BMI [percentile] ^†^ | 98.9 ±1.33 | 98.3 ±2.0 | -0.6 ± 0.2 | < .001 | |  |
| < 90 (normal weight) ^§^ | 0 (0%) | 0 (0%) | - | - | |  |
| >= 90 and < 97 (overweight) [n] ^§^ | 10 (8%) | 21 (18%) | - | - | |  |
| >= 97 and < 99.5 (obesity) [n] ^§^ | 49 (43%) | 52 (45%) | - | - | |  |
| >= 99.5 (extreme obesity) [n] ^§^ | 56 (49%) | 42 (37%) | - | - | |  |
| BMI-SDS ^†^ | 2.55 ± 0.5 | 2.41 ± 0.6 | -0.14 ± 0.1 | < .001 | |  |
| Total fat mass [%] ^†^ | 39.0 ± 6.6 | 37.2 ±6.6 | -1.8 ± 0.9 | < .001 | |  |
| Intraabdominal fat [mm] ^†^ | 45.5 ± 12.5 | 38.7 ± 12.3 | -6.8 ± 1.7 | < .001 | |  |
| **Glucose metabolism** | | | | | | |
| HOMA-IR ^†^ | 5.9 ±3.8 | 5.1 ± 4.2 | -0.8 ± 0.6 | .011 | |  |
| Insulin [µU/ml] ^†^ | 27.8 ± 17.0 | 23.6 ± 16.7 | -4.2 ± 2.3 | .003 | |  |
| C-peptide [ng/ml] ^†^ | 3.5 ± 1.2 | 3.2 ± 1.1 | -0.3 ± 0.2 | .01 | |  |
| Glucose [mmol/l] ^†^ | 4.7 ± 0.3 | 4.8 ± 0.4 | 0.03 ± 0.1 | NS | |  |
| HbA1c [%] ^†^ | 5.3 ± 0.3 | 5.3 ± 0.3 | 0.01 ± 0.04 | NS | |  |
| HbA1c [mmol/mol] ^†^ | 34.6 ± 3.6 | 34.8 ± 3.7 | 0.2 ± 0.5 | NS | |  |
| **Inflammation** | | | | | | |
| CRP [mg/l] ^†^ | 4.5 ± 4.3 | 3.2 ± 3.3 | -1.3 ± 0.6 | .004 | |  |
| Absolute neutrophil count ^†^ | 4.0 ±1.9 | 3.7 ± 1.3 | -0.3 ± 0.3 | NS | |  |
| Leptin [ng/ml] † | 17.0 ± 8.9 | 14.6 ± 8.5 | -2.5 ± 1.3 | | < .001 |  |
| Adiponectin [µg/ml] † | 7.6 ± 2.3 | 8.0 ± 2.3 | -0.5 ± 0.3 | | < .001 |  |

Table S4. Baseline characteristics and change of anthropometric and metabolic parameters during the intervention in patients who underwent OGTT. To rule out differences between patients who underwent OGTT and the whole sample, analyses were carried out in the subset of patients with OGTT. Values obtained were equal to the whole sample (Table 1). Sex and age were equally distributed between males and females from 10 to 14 years. All patients were affected by overweight or obesity, BMI percentile and BMI-SDS were calculated according to reference data from a population in Germany before the obesity epidemic (1). Total fat mass was obtained via bioimpedance measurement, intraabdominal fat measured by ultrasound examination. NS = not significant, § Number of patients and ratio (%), † mean and standard deviation values

| **Female characteristics (n=102)** | | | | |
| --- | --- | --- | --- | --- |
| Age [years] ^†^ | 12 ± 1.4 | | | |
| **Anthropometric parameters** | **Baseline (mean±SD)** | **After intervention (mean±SD)** | **Difference** |  |
| Height [cm] † | 155.9 ± 9.2 | 158.8 ±8.5 | 2.9 ± 1.3 |  |
| Weight [kg] † | 73.6 ± 16.9 | 74.7 ± 16.8 | 1.1 ± 2.5 |  |
| BMI [percentile] † | 98.8 ± 1.4 | 98.3 ± 1.8 | -0.6 ± 0.2 |  |
| < 90 (normal weight) [n] § | 0 (0%) | 1 (1%) | - |  |
| >= 90 and < 97 (overweight) [n] § | 8 (8%) | 20 (20%) | - |  |
| >= 97 and < 99.5 (obesity) [n] § | 49 (48%) | 51 (50%) | - |  |
| >= 99.5 (extreme obesity) [n] § | 45 (44%) | 31 (30%) | - |  |
| BMI-SDS † | 2.5 ± 0.5 | 2.3 ± 0.5 | -0.14 ± 0.1 |  |
| Total fat mass [%] † | 40.1 ± 5.3 | 38.5 ± 5.6 | -1.6 ± 0.8 |  |
| Intraabdominal fat [mm] † | 42.2 ±10.5 | 36.4 ± 9.6 | -5.9 ± 1.5 |  |
| **Glucose metabolism** | | | | |
| HOMA-IR † | 5.6 ± 3.3 | 4.5 ± 2.3 | -1.1 ± 0.4 |  |
| Insulin [µU/ml] † | 26.8 ± 15.0 | 21.6 ± 10.3 | -5.2 ± 1.8 |  |
| C-peptide [ng/ml] † | 3.5 ± 1.1 | 3.2 ± 0.9 | -0.3 ± 0.1 |  |
| Glucose [mmol/l] † | 4.6 ± 0.4 | 4.6 ±0.4 | -0.0 ± 0.1 |  |
| HbA1c [%] † | 5.3 ± 0.3 | 5.3 ± 0.3 | 0.0 ± 0.0 |  |
| HbA1c [mmol/mol] † | 34.2 ± 3.2 | 34.0 ± 3.0 | -0.2 ± 0.5 |  |
| **Inflammation** | | | | |
| CRP [mg/l] † | 3.7 ± 4.0 | 2.5 ± 3.2 | -1.2 ± 0.6 |  |
| Absolute neutrophil count † | 4.2 ± 2.0 | 4.0 ± 1.4 | -0.2 ± 0.3 |  |
| Leptin [ng/ml] † | 16.1 ± 8.2 | 13.1 ± 7.3 | -3.1 ± 1.2 |  |
| Adiponectin [µg/ml] † | 7.5 ± 2.3 | 7.9 ± 2.3 | 0.5 ± 0.4 |  |

Table S5. Baseline characteristics and change of anthropometric and metabolic parameters during the intervention in girls. All patients were affected by overweight or obesity, BMI percentile and BMI-SDS were calculated according to reference data from a population in Germany before the obesity epidemic (1). Total fat mass was obtained via bioimpedance measurement, intraabdominal fat measured by ultrasound examination. § Number of patients and ratio (%), † mean and standard deviation values

| **Male characteristics (n=93)** | | | | |
| --- | --- | --- | --- | --- |
| Age [years] ^†^ | 12 ± 1.4 | | | |
| **Anthropometric parameters** | **Baseline (mean±SD)** | **After intervention (mean±SD)** | **Difference** |  |
| Height [cm] † | 158.0 ± 11.5 | 161.9 ±11.5 | 3.9 ± 1.7 |  |
| Weight [kg] † | 76.6 ± 20.4 | 78.8 ± 20.7 | 2.3 ± 3.1 |  |
| BMI [percentile] † | 98.6 ± 1.6 | 97.8 ± 2.4 | -0.7 ± 0.3 |  |
| < 90 (normal weight) [n] § | 0 (0%) | 1 (1%) | - |  |
| >= 90 and < 97 (overweight) [n] § | 14 (15%) | 21 (23%) | - |  |
| >= 97 and < 99.5 (obesity) [n] § | 47 (41%) | 44 (47%) | - |  |
| >= 99.5 (extreme obesity) [n] § | 32 (34%) | 27 (29%) | - |  |
| BMI-SDS † | 2.4 ± 0.5 | 2.3 ± 0.5 | -0.14 ± 0.1 |  |
| Total fat mass [%] † | 35.4 ± 6.4 | 33.4 ± 6.2 | -2.0 ± 1.0 |  |
| Intraabdominal fat [mm] † | 45.9 ±12.7 | 41.2 ± 13.5 | -4.8 ± 2.0 |  |
| **Glucose metabolism** | | | | |
| HOMA-IR † | 5.4 ± 3.5 | 4.8 ± 4.4 | -0.6 ± 0.6 |  |
| Insulin [µU/ml] † | 25.4 ± 14.9 | 22.2 ± 17.4 | -3.1 ± 2.4 |  |
| C-peptide [ng/ml] † | 3.3 ± 1.2 | 3.0 ± 1.1 | -0.3 ± 0.2 |  |
| Glucose [mmol/l] † | 4.7 ± 0.4 | 4.8 ±0.4 | -0.1 ± 0.1 |  |
| HbA1c [%] † | 5.3 ± 0.3 | 5.3 ± 0.4 | 0.0 ± 0.1 |  |
| HbA1c [mmol/mol] † | 34.0 ± 3.9 | 34.2 ± 4.1 | 0.2 ± 0.6 |  |
| **Inflammation** | | | | |
| CRP [mg/l] † | 3.7 ± 4.0 | 2.5 ± 3.2 | -1.2 ± 0.6 |  |
| Absolute neutrophil count † | 4.2 ± 2.0 | 4.0 ± 1.4 | -0.2 ± 0.3 |  |
| Leptin [ng/ml] † | 13.7 ± 7.6 | 11.2 ± 8.4 | -2.5 ± 1.3 |  |
| Adiponectin [µg/ml] † | 8.0 ± 2.3 | 8.4 ± 2.4 | 0.4 ± 0.4 |  |

Table S6. Baseline characteristics and change of anthropometric and metabolic parameters during the intervention in boys. All patients were affected by overweight or obesity, BMI percentile and BMI-SDS were calculated according to reference data from a population in Germany before the obesity epidemic (1). Total fat mass was obtained via bioimpedance measurement, intraabdominal fat measured by ultrasound examination. § Number of patients and ratio (%), † mean and standard deviation values

| **Parameter** | **BMI-SDS Q_1_** | **BMI-SDS Q_2_** | **BMI-SDS Q_3_** | **BMI-SDS Q_4_** |
| --- | --- | --- | --- | --- |
| Height [cm] | 3.34 (2.04) | 3.25 (2.45) | 3.58 (1.99) | 3.29 (1.96) |
| Weight [kg] | 2.21 (2.14) | 0.41 (2.8) | 1.45 (2.77) | 2.03 (3.98) |
| BMI-SDS | -0.12 (0.05) | -0.21 (0.03) | -0.16 (0.04) | -0.1 (0.06) |
| Total fat mass [%] | -1.52 (0.94) | -2.1 (0.95) | -1.72 (0.93) | -1.84 (1.23) |
| Intraabdominal fat [mm] | -3.64 (2) | -2.56 (2.02) | -6.86 (2.45) | -7.72 (2.5) |
| HOMA-IR | -0.79 (0.47) | -0.92 (0.48) | -1.37 (0.64) | -0.36 (0.98) |
| Insulin [µU/ml] | -3.59 (2.04) | -4.41 (2.16) | -6.6 (2.91) | -2.18 (3.92) |
| C-peptide [ng/ml] | -0.32 (0.19) | -0.28 (0.18) | -0.46 (0.2) | -0.24 (0.23) |
| Glucose [mmol/l] | 0 (0.09) | 0.01 (0.08) | 0.03 (0.07) | 0.01 (0.07) |
| HbA1c [%] | 0.02 (0.06) | 0.01 (0.07) | 0 (0.08) | -0.03 (0.05) |
| HbA1c [mmol/mol] | 0.18 (0.64) | 0.1 (0.76) | -0.02 (0.87) | -0.28 (0.63) |
| CRP [mg/l] | -0.24 (0.63) | -1.93 (0.72) | -1.18 (0.94) | -0.74 (0.92) |
| Absolute neutrophil count | 0.01 (0.36) | -0.56 (0.44) | -0.11 (0.23) | 0.16 (0.33) |
| Leptin [ng/ml] | -2.83 (0.98) | -3.2 (1.07) | -3.31 (2.02) | -1.79 (2.07) |
| Adiponectin [µg/ml] | 0.85 (0.62) | 0.02 (0.59) | 0.67 (0.45) | 0.29 (0.49) |

Table S7. Differences in anthropometric and metabolic parameters before and after the intervention by BMI-SDS quartile before intervention. Patients were divided in quartiles on the basis of BMI-SDS before intervention, BMI-SDS Q1 is the quarter of patients with the greatest BMI-SDS before intervention. For each parameter, differences of the means and standard deviation within that quartile is given. BMI-SDS was calculated according to reference data from a population in Germany before the obesity epidemic (1). Total fat mass was obtained via bioimpedance measurement, intraabdominal fat measured by ultrasound examination.

|  | | | | | |
| --- | --- | --- | --- | --- | --- |
| **Estimated nutrient intake** | **Baseline (mean±SD)** | **After intervention (mean±SD)** | **Difference** | **p value** |  |
| Daily intake [kcal] | 2155 ± 1252 | 1533 ± 908 | -623 ± 118 | < .001 |  |
| Carbohydrates [g] | 264 ± 165 | 190 ± 118 | -75 ± 15 | < .001 |  |
| Proteins [g] | 75 ± 45 | 59 ± 38 | -15 ± 4 | < .001 |  |
| Fat [g] | 85 ± 54 | 56 ± 38 | -29 ± 5 | < .001 |  |
| Fiber [g] | 22 ± 17 | 21 ± 15 | -1 ± 2 | NS |  |

Table S8. Differences in estimated nutrient intake before and after intervention. Detailed dietary intake was recorded using the validated food frequency questionnaire "What do you eat?" from the German National Health Interview and Examination Survey for Children and Adolescents (2) before and after intervention. Intake of calories was calculated by multiplying estimated amount of foods from the questionnaire with average amounts of macronutrients and calories for each food type. NS = not significant

**Supplementary Figures**


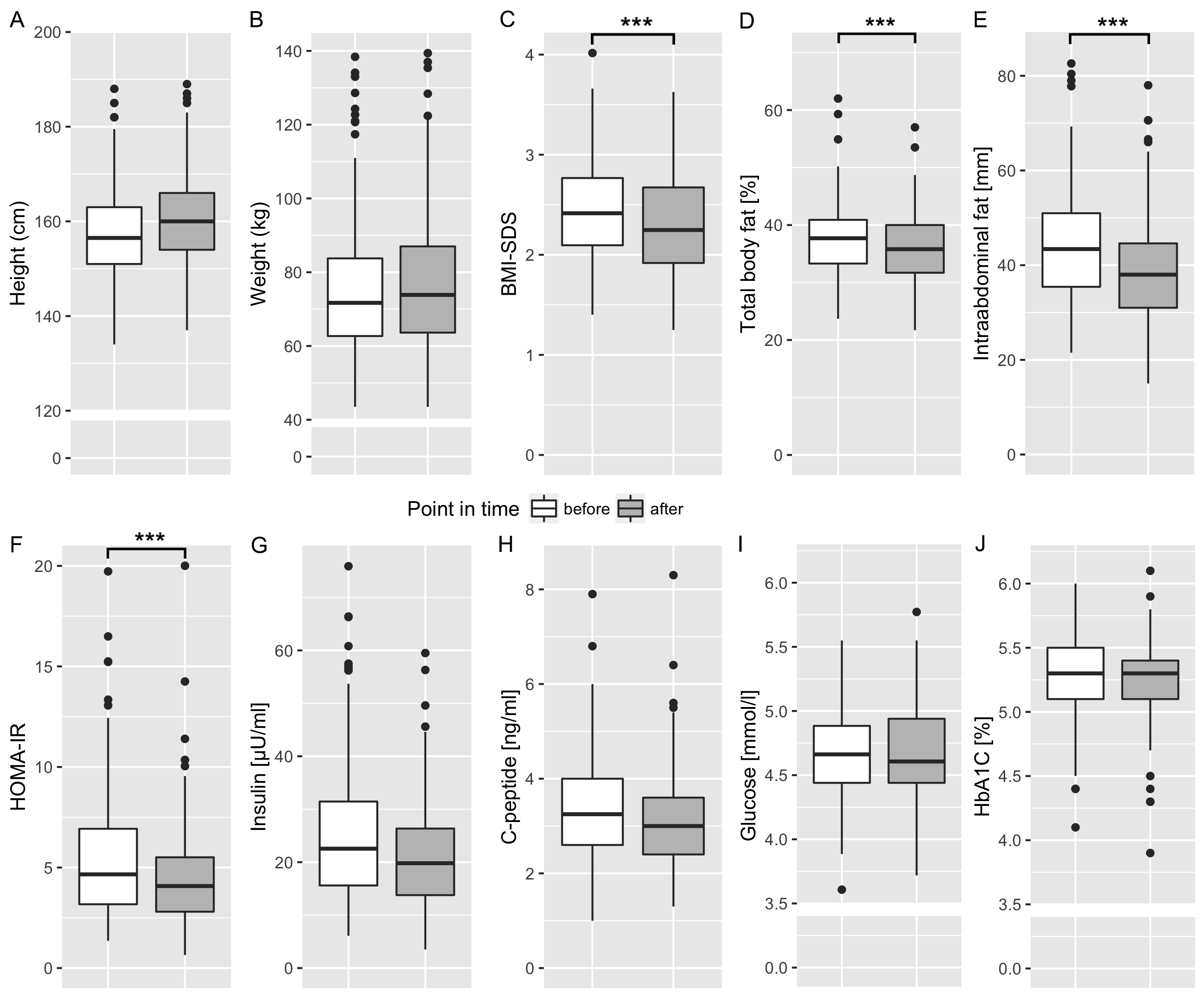


Fig S1. Body composition and glucose metabolism parameters before (white) and after (gray) intervention. Body composition parameters height (A), weight (B), BMI-SDS (C), total body fat (D) and intraabdominal fat (E). Despite weight showing an upwards trend, increase in height was greater to show an overall significant reduction of BMI-SDS, total body fat and intraabdominal body fat. Glucose metabolism improved significantly, as shown by HOMA-IR (F), Insulin (G), C-Peptide (H), while fasting glucose (I) and HbA1c (J) were both before and after within the reference range. *** p < 0.001


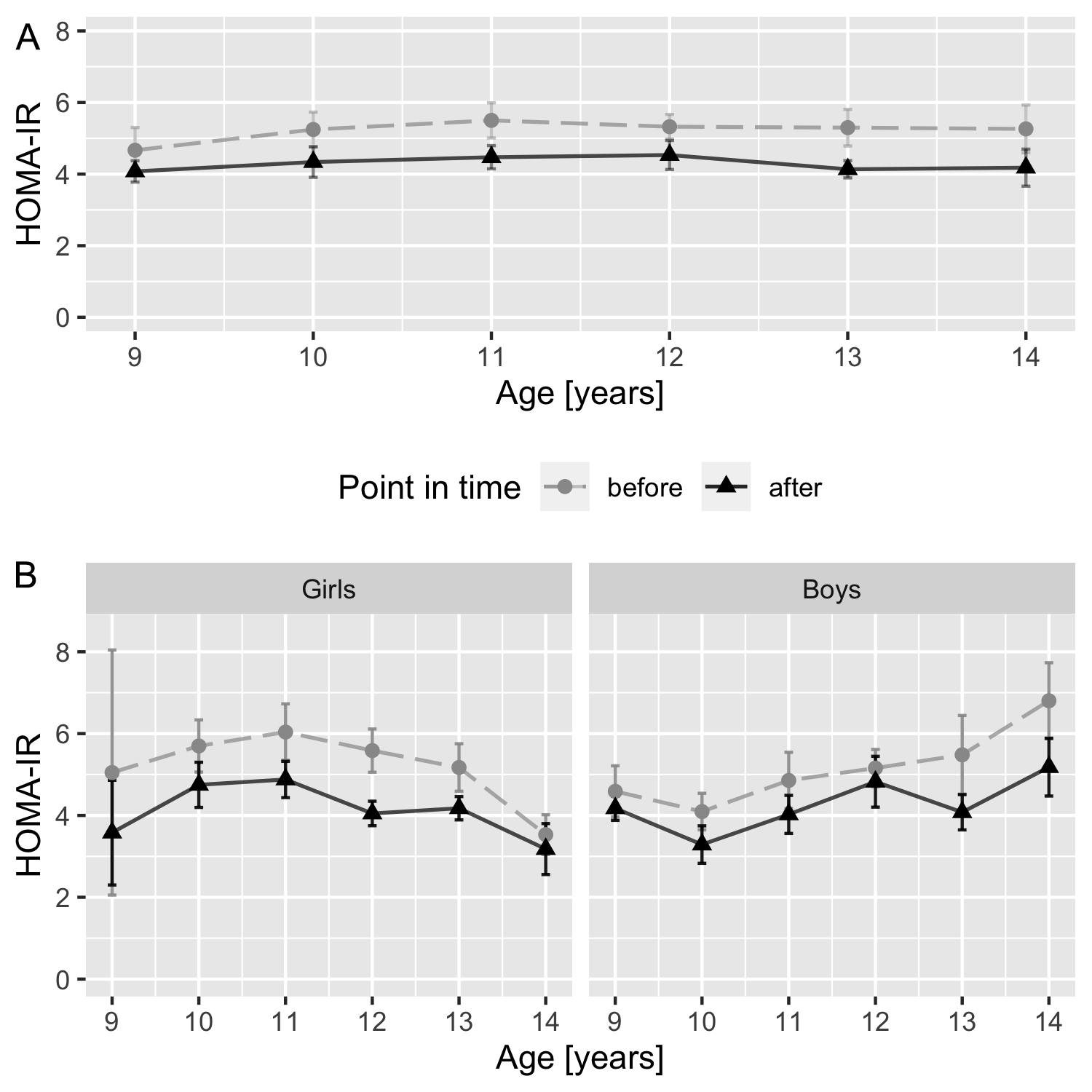


Fig S2. Mean HOMA-IR levels (± standard error of the mean) before and after intervention for all patients (A) and by gender (B) for age at inclusion. Over all patients, insulin resistance depicted by HOMA-IR shows a decrease for every age. The overall descending trend of the IR in boys and ascending trend in girls is consistent with the physiologic course of IR in puberty, having its peak at age 13 in boys and age 15 in girls with subsequent descent and might be independent to obesity (3). In this graph, three outliers were removed.


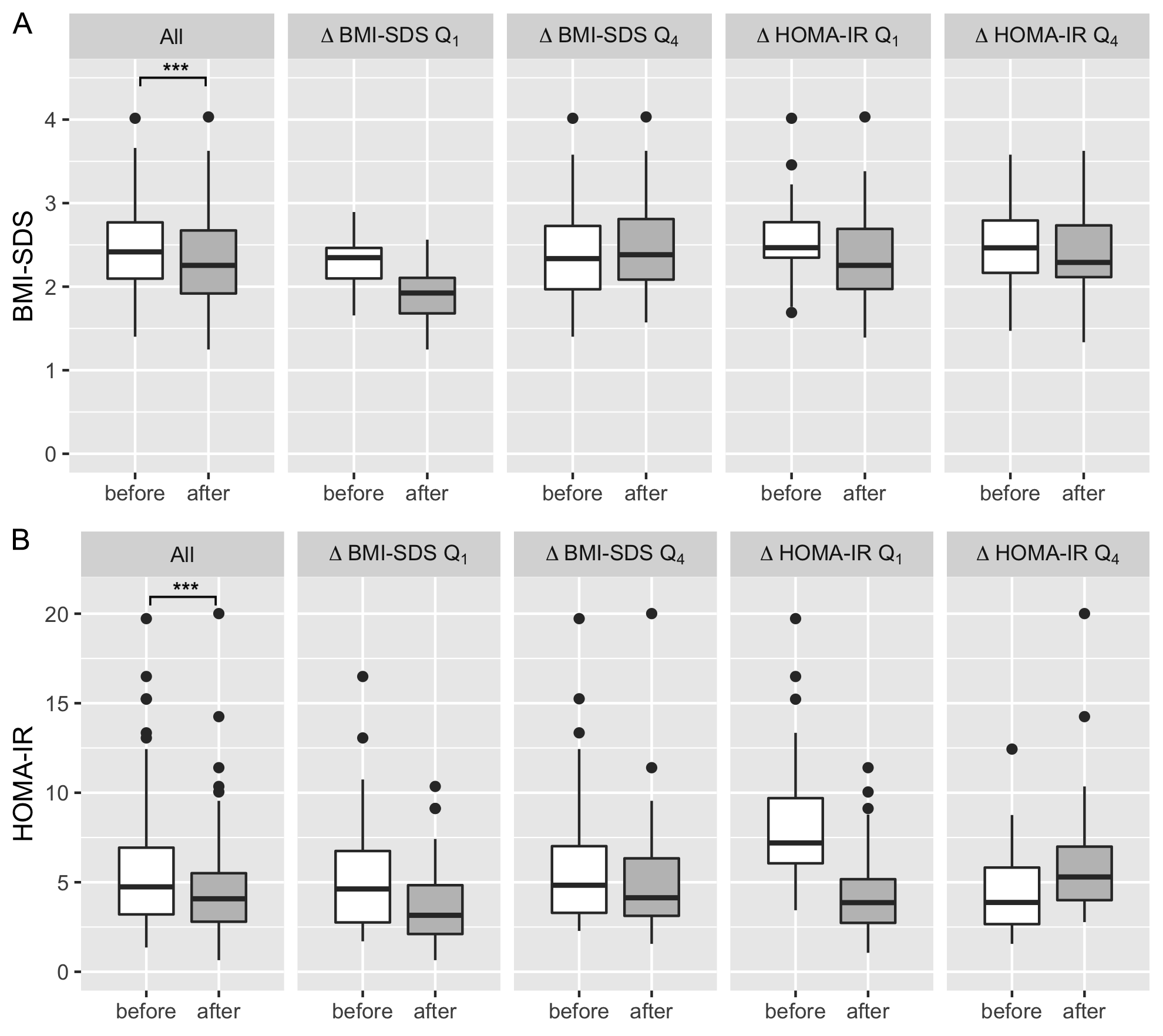


Fig S3. BMI-SDS (A) and HOMA-IR (B) before and after intervention in quartiles of Δ BMI-SDS and Δ HOMA‑IR. A: BMI-SDS in all patients (All) and in the first (Q_1_, decrease) and last (Q_4_, increase) quartile of Δ BMI-SDS and Δ HOMA-IR. B: HOMA-IR in all patients (All) and in the first (Q_1_, decrease) and last (Q_4_, increase) quartile of Δ BMI-SDS and Δ HOMA-IR. Both parameters decreased independently in the whole study population. *** p < 0.001


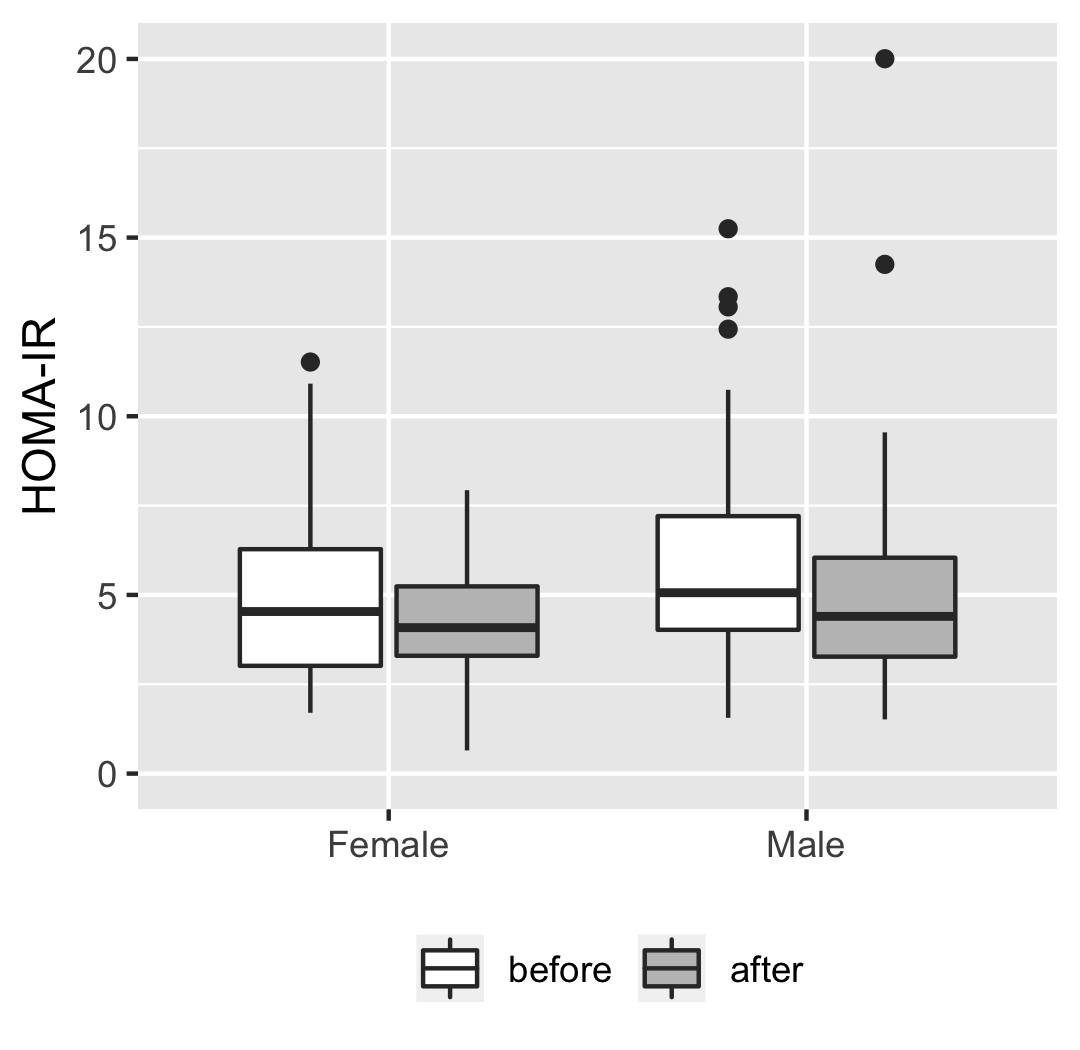


Fig S4. HOMA-IR levels in postpubertal boys and girls before and after intervention. To rule out a potential confounding effect of puberty, effects of the intervention program on HOMA-IR were assessed in patients who were after onset of puberty before and after intervention. Mean HOMA-IR levels before/after intervention are shown as boxplots: on the left in the subgroup of girls who had menarche already before intervention (n=44); on the right in the subgroup of boys with SHBG values < 50nmol/l before intervention (n=69), indicating Tanner stage II (4). Wilcoxon signed-rank test yielded p < 0.05 for both.


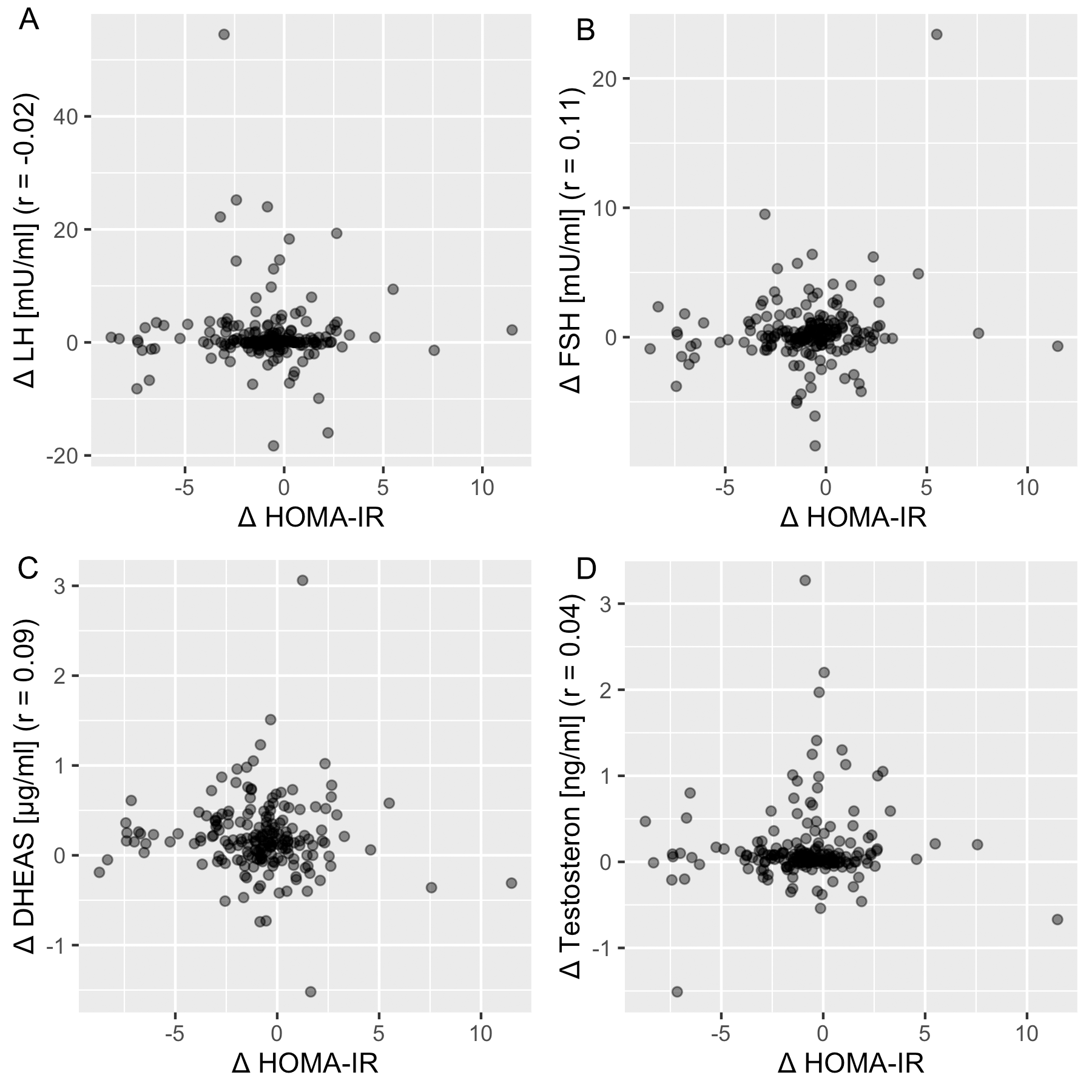


Fig S5. Correlation of insulin resistance index HOMA-IR with sex hormones. Luteinizing hormone (LH), follicle-stimulating hormone (FSH), dehydroepiandrosterone sulfat (DHEAS), and testosterone are shown in subfigures A, B, C, D, respectively.

**Supplementary References**

1. Kromeyer-Hauschild K WM, Kunze D et al. Percentile für den Body Mass Index für das Kindes- und Jugendalter unter Heranziehung verschiedener deutscher Stichproben. Monatsschrift Kinderheilkunde. 2001;149:807-18.

2. Truthmann J, Mensink GB, Richter A. Relative validation of the KiGGS Food Frequency Questionnaire among adolescents in Germany. Nutr J. 2011;10:133. doi: 10.1186/1475-2891-10-133. PubMed PMID: 22152115; PubMed Central PMCID: PMCPMC3261099.

3. Shashaj B, Luciano R, Contoli B, Morino GS, Spreghini MR, Rustico C, et al. Reference ranges of HOMA-IR in normal-weight and obese young Caucasians. Acta Diabetol. 2015. doi: 10.1007/s00592-015-0782-4. PubMed PMID: 26070771.

4. Kim MR, Gupta MK, Travers SH, Rogers DG, Van Lente F, Faiman C. Serum prostate specific antigen, sex hormone binding globulin and free androgen index as markers of pubertal development in boys. Clin Endocrinol (Oxf). 1999;50(2):203-10. Epub 1999/07/09. doi: 10.1046/j.1365-2265.1999.00636.x. PubMed PMID: 10396363.
